# Mental illness, challenging behaviour, and psychotropic drug prescribing in people with intellectual disability: UK population based cohort study

**DOI:** 10.1136/bmj.h4326

**Published:** 2015-09-01

**Authors:** Rory Sheehan, Angela Hassiotis, Kate Walters, David Osborn, André Strydom, Laura Horsfall

**Affiliations:** 1 Division of Psychiatry, University College London, London W1T 7NF, UK; 2 Research Department of Primary Care and Population Health, University College London (Royal Free Campus), London NW3 2PF, UK

## Abstract

**Objectives** To describe the incidence of recorded mental illness and challenging behaviour in people with intellectual disability in UK primary care and to explore the prescription of psychotropic drugs in this group.

**Design** Cohort study.

**Setting** 571 general practices contributing data to The Health Improvement Network clinical database.

**Participants** 33 016 adults (58% male) with intellectual disability who contributed 211 793 person years’ data.

**Main outcome measures** Existing and new records of mental illness, challenging behaviour, and psychotropic drug prescription.

**Results** 21% (7065) of the cohort had a record of mental illness at study entry, 25% (8300) had a record of challenging behaviour, and 49% (16 242) had a record of prescription of psychotropic drugs. During follow-up, the rate of new cases of mental illness in people without a history at cohort entry was 262 (95% confidence interval 254 to 271) per 10 000 person years and the rate of challenging behaviour was 239 (231 to 247) per 10 000 person years. The rate of new psychotropic drug prescription in those without a previous history of psychotropic drug treatment was 518 (503 to 533) per 10 000 person years. Rates of new recording of severe mental illness declined by 5% (95% confidence interval 3% to 7%) per year (P<0.001), and new prescriptions of antipsychotics declined by 4% (3% to 5%) per year P<0.001) between 1999 and 2013. New prescriptions of mood stabilisers also decreased significantly. The rate of new antipsychotic prescribing was significantly higher in people with challenging behaviour (incidence rate ratio 2.08, 95% confidence interval 1.90 to 2.27; P<0.001), autism (1.79, 1.56 to 2.04; P<0.001), and dementia (1.42, 1.12 to 1.81; P<0.003) and in those of older age, after control for other sociodemographic factors and comorbidity.

**Conclusions** The proportion of people with intellectual disability who have been treated with psychotropic drugs far exceeds the proportion with recorded mental illness. Antipsychotics are often prescribed to people without recorded severe mental illness but who have a record of challenging behaviour. The findings suggest that changes are needed in the prescribing of psychotropics for people with intellectual disability. More evidence is needed of the efficacy and safety of psychotropic drugs in this group, particularly when they are used for challenging behaviour.

## Introduction

Approximately 1% of the population have intellectual disability, defined as a significant deficit in cognitive and adaptive function with onset during the developmental period.^1^ People with intellectual disability develop mental illness at rates similar to or higher than the general population,^2^ but atypical presentations, deficits in communication and health literacy, and difficulties in accessing services might mean that mental illness in people with intellectual disability is under-recorded. In addition, a significant proportion of people with intellectual disability display challenging behaviour, also known as behaviour that challenges or problem behaviours—that is, behaviour of an intensity, frequency, or duration that threatens the physical safety of the person or others or restricts access to community facilities.^3^

Concern has existed for many years that psychotropic drugs in general and antipsychotics in particular are overused in people with intellectual disability and might often be prescribed for challenging behaviour in itself rather than for diagnosed mental illness, despite lack of evidence of efficacy.^4^ ^5^ ^6^ ^7^ However, obtaining accurate estimates of the rates of psychotropic drug use in this group has been difficult, and the applicability of existing literature is limited by inconsistent definitions of intellectual disability and use of small or highly selected samples. Despite the evident interest in this topic from policy and care perspectives,^8^ no comprehensive examination of psychotropic drug use in adults with intellectual disability in UK primary care has been carried out, and findings from other countries may not be generalisable given differences in healthcare practice and provision.

Evidence suggests that use of psychotropic drugs in the general population has increased consistently over the past several years,^9^ ^10^ but little research has explored longitudinal trends in prescribing of psychotropics to people with intellectual disability. Whether deinstitutionalisation, increased awareness of drugs’ side effects, and attempts to reduce inappropriate drug treatment in people with intellectual disability by the introduction of prescribing guidelines have changed prescribing practice remains unclear.^11^

Using a very large and representative sample of anonymised primary healthcare records from the United Kingdom, we have described the rates of recorded mental illness, challenging behaviour, and use of psychotropic drugs in people with intellectual disability in the primary care setting. We also report the patterns of prescribing of psychotropics over the past 15 years and the factors associated with prescription of antipsychotics.

## Methods

### Data source

The Health Improvement Network (THIN) is a large primary care database that contains the electronic health records of more than 3.7 million active patients in 571 general practices (www.epic-uk.org/our-data/our-data.shtml). The patients included are representative of the UK population as a whole in terms of age, sex, medical conditions, and death rates.^12^ ^13^ Data from THIN are anonymised at source and collated by IMS Health before being made available for research. 

Ninety eight per cent of the UK population are registered with a general practitioner, who acts as the gatekeeper to healthcare services for all patients, regardless of level of disability or living arrangements.^14^ General practitioners can refer complex cases to hospital based specialists, such as psychiatrists, who make an assessment and offer advice on further investigation or management. If drug treatment is indicated, this is usually prescribed by general practitioners, who hold prescribing budgets. Clinicians or practice staff enter information from the general practice consultation or secondary care specialist into a computerised system as Read codes, standardised clinical terms based on a hierarchical system.^15^ The general practice record thus contains a comprehensive and accurate longitudinal record of a person’s clinical encounters and their outcomes.

The THIN database contains symptoms, diagnoses, referrals to secondary care, and a record of treatments and of prescriptions, which are classified according to chapters in the *British National Formulary*. THIN is well suited to studying drug prescription, as clinicians must code the prescribed drug before the prescription can be issued, so all prescriptions issued in primary care are recorded. Recording of illness, including for mental and developmental disorders, has been shown to be accurate in electronic primary care records, and estimates of disease prevalence closely approximate those determined by other means, making the data suitable for epidemiological research.^16^ Patients’ demographic information and a measure of social deprivation (the Townsend score), which is a composite score in fifths based on Census recording of unemployment, car ownership, home ownership, and overcrowding, is also recorded.^17^

### Study cohort

The study cohort included adults with intellectual disability drawn from UK primary care. General practitioners have been incentivised to keep a register of all patients with known intellectual disability since 2007 as part of the quality and outcomes framework (QOF) scheme (http://qof.hscic.gov.uk/). We used established methods to create our intellectual disability code list,^18^ which was based on that used in a previous study.^19^ We included people with a QOF intellectual disability code as well as those who were not identified by the QOF scheme but who had a Read code anywhere in their record signalling intellectual disability or a condition associated with intellectual disability in more than 50% cases. This has been shown to substantially increase the detection of people with intellectual disability in the THIN database.^19^ Before extracting the cohort for this study and applying the age exclusions, we used the code list to estimate the prevalence of intellectual disability in the 3.7 million active patients in THIN. We identified 32 306 active adults with intellectual disability, or 0.9%, which is comparable to population prevalence estimates of intellectual disability at 1%.^20^

People in the cohort contributed different lengths of follow-up to the study. The date of entry into the cohort for each person was taken as the latest of the date of registration at a practice contributing to THIN plus one year to account for the transfer of historical information,^21^ the year they turned 18, the start of the study period (1 January 1999), and the date their general practice was recording data consistent with the two key indicators that help to affirm the quality of the entered data (acceptable mortality reporting and acceptable computer usage).^22^ ^23^ The end date for each person was the earliest of the date that they left the practice, the date they turned 100 years old, or the end of the study period (31 December 2013). We excluded people who contributed less than 12 months’ data to THIN, as these records often include temporary and visiting patients for whom the quality of data recording is poor.

### Outcomes of interest

#### Neuropsychiatric diagnoses

We interrogated the record of each person who met the inclusion criteria for Read codes for mental illness. We divided mental illness into severe mental illness (schizophrenia, bipolar disorder, other psychosis), depression (including mixed depression-anxiety), and anxiety. If a person had a record of two of the disorders within the severe mental illness category (such as a code for schizophrenia and another for bipolar disorder), for the purpose of describing the case mix at cohort entry we used a hierarchical system whereby the schizophrenia diagnosis was taken in preference to bipolar disorder, and this in turn was used in preference to the group of “other psychosis.” We also included code lists for dementia, autism, and epilepsy to examine the relation with challenging behaviour and prescribing of antipsychotic drugs. We determined neuropsychiatric diagnoses by screening people’s records for relevant codes that our team has used to identify these conditions in previous studies. For conditions that are generally present from a young age, in this case autism and epilepsy, we took a code entered anywhere in the record as evidence of the condition at cohort entry. Mental illness, epilepsy, and dementia are also included in the QOF scheme and should be reliably recorded by general practitioners.

#### Challenging behaviour

We defined challenging behaviour with reference to the National Institute for Health and Care Excellence’s conceptualisation of the term and included the following behaviours: aggression, self injury, stereotypic behaviour, agitation, disruptive or destructive acts, withdrawn behaviour, arson, and sexual misconduct.^24^ We constructed a list of Read codes describing these behaviours by screening the full Read code dictionary for potentially suitable terms. After discussion and on the basis of clinical experience of managing patients with intellectual disability and knowledge of the literature, we also included codes for sleep disturbance.^25^ The list was then refined in an iterative process through consultation between four clinical academics (three psychiatrists specialising in intellectual disability and one general practitioner); the final list contained more than 200 codes (web appendix A). We recorded people as having a history of challenging behaviour at cohort entry if at least one of any of these codes was present in their record and recorded them as having incident challenging behaviour if a code was added during follow-up. To assess the performance of the challenging behaviour code list, we examined the variable in relation to factors known to be associated with challenging behaviour in people with intellectual disability, including the presence of other neuropsychiatric diagnoses. We also examined the challenging behaviour code list in relation to severity of intellectual disability in the subset for which this information was coded as mild, moderate, severe, or profound and also compared recording of challenging behaviour in people with Down’s syndrome with those with autism.

#### Prescription of psychotropic drugs

We divided psychotropic drugs into several classes according to *British National Formulary* sub-chapter: antipsychotics (including first and second generation agents), antidepressants, mood stabilisers, anxiolytics and hypnotics (including benzodiazepines), antidementia drugs, and drugs for attention-deficit/hyperactivity disorder.

### Statistical analysis

We used multivariable mixed Poisson regression to examine time trends of recording of mental illness and new prescriptions for psychotropic drugs in people without a history at cohort entry and to calculate incidence rate ratios adjusted for any temporal changes in age and sex. We included general practice as a random effect to account for any data clustering. If people received a new prescription for a drug after cohort entry (that is, during follow-up) we considered them to be no longer at risk for that drug prescription and removed them from the cohort. To examine the possibility of non-linear time trends, year was initially modelled as a continuous variable and we then used the likelihood ratio test to compared this with a model in which year was entered as a categorical variable. A significant test result suggests that the categorical model is a better fit to the data and a linear time trend assumption may not be appropriate.

We also used multivariable mixed Poisson regression to examine factors associated with new records of challenging behaviour and new prescription of antipsychotic drugs in people without a history at cohort entry. We investigated multiplicative interactions between sex, challenging behaviour, and prescription of antipsychotics as a pre-specified hypothesis by including the appropriate interaction terms in the regression model. In the regression model examining new prescriptions of antipsychotics, we tested the influence of excluding sleep codes from the challenging behaviour code list as a sensitivity analysis. We used Wald tests to assess overall significance for categorical variables and categorical interaction terms. We considered a P value of 0.05 to be statistically significant (two tailed) and used Stata version 13 for all analyses.

### Patient involvement

No patients were involved in setting the research question or the outcome measures; nor were they involved in the design and implementation of the study. We plan to work with our local service user research group (the Camden Advocacy Project) to prepare accessible (easy read) versions of the abstract and manuscript content, which will be widely disseminated.

## Results

### Characteristics of cohort

In total, 33 016 people with a record of intellectual disability met the inclusion criteria. Table 1 gives the baseline characteristics of the cohort. Fifty eight per cent were male, and the average age at study entry was 36.3 years. Median follow-up time was 5.5 (interquartile range 2.2-11.5) years. Overall, 211 793 person years’ data were collected.

**Table 1 tbl1:** Characteristics of cohort at study entry. Values are numbers (percentages) unless stated otherwise

Characteristic	Value (n=33 016)
Mean (SD) age, years	36.3 (16.4)
Median (interquartile range) follow-up time, years	5.5 (2.2-11.5)
Male sex	19 139 (58)
Autism	4925 (15)
Epilepsy	7517 (23)
Dementia	319 (1)
History of mental illness	7065 (21)
Severe mental illness	2364 (7)
Schizophrenia	1313 (4)
Bipolar disorder	439 (1)
Psychosis, other	612 (2)
Depression (including mixed anxiety-depression)	3620 (11)
Anxiety	1845 (6)
History of challenging behaviour	8300 (25)
History of psychotropic drug prescription	16 242 (49)
Antipsychotic	7028 (21)
Antidepressant	6614 (20)
Mood stabiliser	6698 (20)
Anxiolytic/hypnotic	7152 (22)
Antidementia drugs	254 (0)
Drugs for attention-deficit/hyperactivity disorder	623 (2)

### Recording of mental illness

In total, 21% of the cohort had a record of mental illness at study entry, including 7% with a record of severe mental illness (schizophrenia 4%, bipolar disorder 1%, other psychosis 2%) (table 1). The incidence of new records of mental illness was 262 (95% confidence interval 254 to 271) per 10 000 person years during follow-up (table 2). New records of depression were most frequently recorded. By the end of the study period, the proportion of people with a record of mental illness (not including those with only challenging behaviour) at any point in their primary care notes was 34% (7065 at baseline and 3998 during follow-up), and the proportion with a record of severe mental illness was 9% (tables 1 and 2).

**Table 2 tbl2:** Incidence of recorded mental illness, challenging behaviour, and psychotropic drug prescription in adults with intellectual disability in UK primary care, 1999-2013

Variable	No of events	No of patient years (×10 000)	Incidence per 10 000 patient years (95% CI)
Any mental illness	3998	15.2	262 (254 to 271)
Severe mental illness	617	19.5	32 (29 to 34)
Depression	3054	17.9	171 (165 to 177)
Anxiety	2512	18.0	139 (134 to 145)
Challenging behaviour	3615	15.1	239 (231 to 247)
Any psychotropic drug	4640	9.0	518 (503 to 533)
Antipsychotic	2107	15.9	132 (127 to 138)
Antidepressant	4733	15.1	313 (305 to 323)
Mood stabiliser	1337	16.3	82 (78 to 87)
Anxiolytic/hypnotic	4835	14.7	330 (321 to 339)
Antidementia drugs	204	21.1	10 (8 to 11)
Drugs for attention-deficit/hyperactivity disorder	52	21.0	2 (2 to 3)

Analysis of time trends in the recording of severe mental illness showed a gradual decline in the rate of recording by 5% (95% confidence interval 3% to 7%) per year between 1999 and 2013 (P<0.001), after adjustment for changes in age and sex of the cohort (fig 1). Rates of recording of depression and anxiety did not change.

**Figure fig1:**
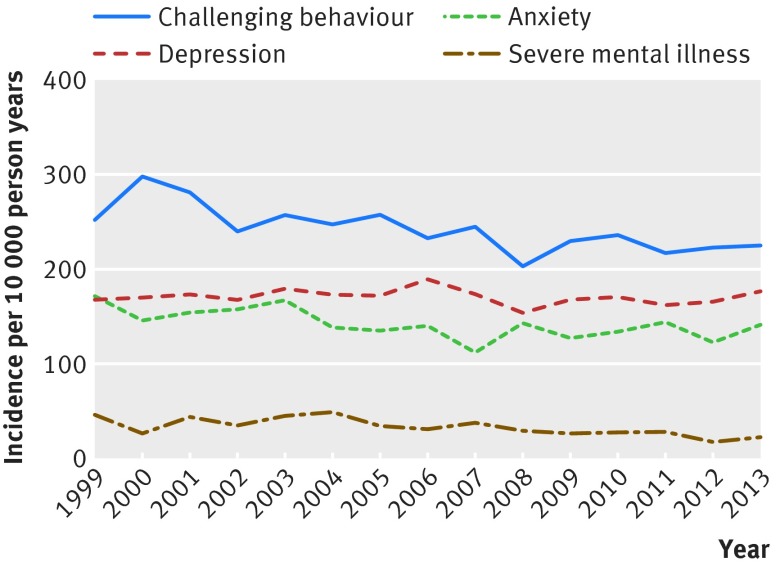
**Fig 1** Time trends in new recording of mental illness and challenging behaviour in adults with intellectual disability in UK primary care, 1999-2013

### Challenging behaviour

In total, 8300 (25%) people had a history of challenging behaviour at study entry. The rate of new cases of challenging behaviour during follow-up in those without a previous history at cohort entry was 239 (231 to 247) per 10 000 person years. By the end of the study period, 36% of people in the cohort had a record of challenging behaviour in their electronic health record (8300 at baseline and 3615 during follow-up) (tables 1 and 2). The most common Read codes in the challenging behaviour code list found in the cohort were “behavioural problem,” “behaviour disorder,” “behavioural problems,” “agitated,” and “agitated—symptom.” Codes describing specific behaviours were used less frequently. Excluding sleep codes reduced the number with challenging behaviour at baseline to 7531 and the rate of new records during follow-up to 175 (169 to 182) per 10 000 person years.

The median number of Read codes for challenging behaviour in people with a record of challenging behaviour (including sleep codes) was 2 (interquartile range 1-4). New records of challenging behaviour were significantly more common in people older than 50 years and in those with mental illness (including severe mental illness, depression, and anxiety), autism, dementia, and epilepsy (table 3). We found no association of new recording of challenging behaviour with sex or social deprivation. New recording of challenging behaviour showed a non-significant downward trend during the study period (fig 1). In separate analyses, we identified a positive association between challenging behaviour and degree of intellectual disability in the 5332 people for whom information on severity had been coded; challenging behaviour was three times more common in people with profound intellectual disability than in those described as having mild intellectual disability (incidence rate ratio 2.97 (95% confidence interval 2.09 to 4.21; P<0.001); data not shown). Compared with people with Down’s syndrome, those coded as having autism were more than twice as likely to be coded as having challenging behaviour (incidence rate ratio 2.15, 1.87 to 2.48; p<0.001; data not shown).

**Table 3 tbl3:** Associations with new recording of challenging behaviour in adults with intellectual disability in UK primary care, 1999-2013

Factor	Rate (per 10 000 patient years) (95% CI)	Incidence rate ratio* (95% CI)	P value
Sex:			
Male	243 (231 to 255)	1 (reference)	NS
Female	235 (225 to 246)	0.97 (0.91 to 1.04)	
Age group, years:			
18-29	218 (204 to 234)	1 (reference)	<0.001
30-39	220 (204 to 237)	1 (0.90 to 1.11)	
40-49	226 (211 to 242)	1.01 (0.91 to 1.12)	
50-59	260 (241 to 282)	1.13 (1.02 to 1.26)	
60-69	286 (259 to 316)	1.27 (1.12 to 1.45)	
70-79	304 (262 to 351)	1.40 (1.18 to 1.65)	
≥80	382 (302 to 483)	1.78 (1.38 to 2.30)	
Townsend deprivation fifth:			
1 (least deprived)	230 (212 to 251)	1 (reference)	NS
2	247 (229 to 267)	1.06 (0.95 to 1.20)	
3	226 (210 to 244)	1.02 (0.90 to 1.14)	
4	225 (210 to 242)	0.97 (0.87 to 1.10)	
5 (most deprived)	264 (246 to 282)	1.10 (0.98 to 1.25)	
Missing	247 (212 to 288)	1.08 (0.89 to 1.32)	
Neuropsychiatric diagnosis:			
No severe mental illness	224 (216 to 232)	1 (reference)	<0.001
Severe mental illness	442 (403 to 484)	1.69 (1.53 to 1.88)	
No depression	201 (193 to 209)	1 (reference)	<0.001
Depression	399 (376 to 423)	1.71 (1.58 to 1.86)	
No anxiety	209 (201 to 217)	1 (reference)	<0.001
Anxiety	384 (360 to 409)	1.44 (1.33 to 1.57)	
No autism	227 (219 to 235)	1 (reference)	<0.001
Autism	380 (346 to 418)	1.83 (1.64 to 2.03)	
No dementia	233 (225-240)	1 (reference)	<0.001
Dementia	482 (416 to 558)	1.71 (1.45 to 2.01)	
No epilepsy	225 (217 to 234)	1 (reference)	<0.001
Epilepsy	287 (270 to 306)	1.41 (1.30 to 1.52)	

NS=not significant.

*Adjusted for age, sex, Townsend deprivation fifth, neuropsychiatric diagnoses, and year of study entry.

### Prescription of psychotropic drugs

A history of prescription of psychotropic drugs was present in 49% of the cohort at study entry, and 63% had a record by the end of data collection. The incidence rate of new prescription of any psychotropic drugs over the follow-up period was 518 (503 to 533) per 10 000 person years. The most common class of drugs to be prescribed was anxiolytics/hypnotics, followed by antidepressants, antipsychotics, and mood stabilisers. The rate of prescription of antidementia drugs and drugs for attention-deficit/hyperactivity disorder was much lower at 10 (8 to 11) per 10 000 person years and 2 (2 to 3) per 10 000 person years, respectively, which precluded further investigation of time trends in their prescription.

Prescription of antipsychotics fell by 4% (3% to 5%; P<0.001) per year over the course of the study period, and the prescription of mood stabilisers also decreased by 4% (3% to 6%; P<0.001) per year. We found no significant time trends in the prescription of other classes of psychotropic drugs (fig 2). Prescription of antidepressants fell significantly in 2005, but rates of prescription in 2013 were similar to those seen at the start of the observation period in 1999.

**Figure fig2:**
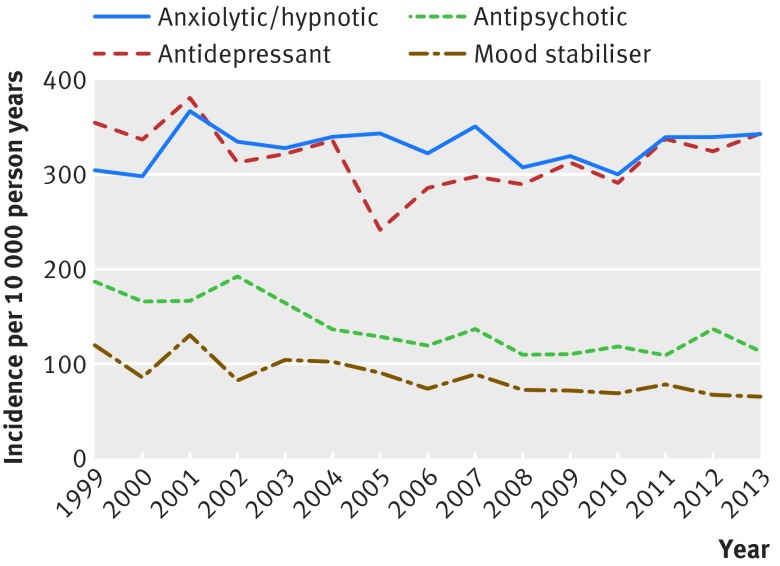
**Fig 2** Time trends in new prescriptions of psychotropic drugs in adults with intellectual disability in UK primary care, 1999-2013

New prescriptions for antipsychotics were significantly more common in older people and in those with a record of challenging behaviour, severe mental illness, depression, anxiety, autism, dementia, and epilepsy (tables 4 and 5). We found no association of new prescription of antipsychotics with sex or level of social deprivation. People with a record of challenging behaviour were more than twice as likely to receive a prescription for antipsychotics compared with those without a record of challenging behaviour (incidence rate ratio 2.08, 1.90 to 2.27; P<0.001) after control for neuropsychiatric diagnoses. Excluding sleep codes from the definition of challenging behaviour as a sensitivity analysis increased the magnitude of the association with prescribing of antipsychotics (incidence rate ratio 3.49, 3.19 to 3.82; P<0.001).

**Table 4 tbl4:** Incidence of new prescription of antipsychotic drugs in adults with intellectual disability in UK primary care, 1999-2013, by sociodemographic factors and neuropsychiatric diagnosis

Factor	Rate (per 10 000 patient years) (95% CI)
Sex:	
Male	134 (125 to 142)
Female	131 (124 to 139)
Age group, years:	
18-29	130 (120 to 141)
30-39	122 (111 to 134)
40-49	118 (107 to 130)
50-59	132 (118 to 147)
60-69	149 (129 to 173)
70-79	213 (177 to 257)
≥80	305 (236 to 394)
Townsend deprivation fifth:	
1 (least deprived)	131 (118 to 147)
2	135 (121 to 149)
3	136 (124 to 149)
4	133 (122 to 146)
5 (most deprived)	122 (110 to 134)
Missing	154 (127 to 186)
Neuropsychiatric diagnosis:	
Challenging behaviour	237 (222 to 252)
No challenging behaviour	96 (90 to 101)
Severe mental illness	1005 (890 to 1135)
No severe mental illness	118 (112 to 123)
Depression	250 (233 to 269)
No depression	104 (99 to 110)
Anxiety	246 (228 to 265)
No anxiety	109 (104 to 115)
Autism	232 (207 to 259)
No autism	123 (118 to 129)
Dementia	257 (208 to 318)
No dementia	129 (124 to 135)
Epilepsy	138 (126 to 150)
No epilepsy	131 (124 to 137)

**Table 5 tbl5:** Associations with new antipsychotic drug prescription in adults with intellectual disability in UK primary care, 1999-2013

Factor	IRR* (95% CI)	P value	IRR† (95% CI)	P value
Sex:				
Male	1 (reference)	NS	1 (reference)	NS
Female	1.04 (0.95 to 1.12)		1.01 (0.93 to 1.11)	
Age group, years:				
18-29	1 (reference)	<0.001	1 (reference)	<0.001
30-39	0.95 (0.84 to 1.07)		0.99 (0.88 to 1.13)	
40-49	0.91 (0.80 to 1.03)		0.96 (0.85 to 1.10)	
50-59	1.01 (0.88 to 1.16)		1.08 (0.93 to 1.25)	
60-69	1.14 (0.97 to 1.35)		1.32 (1.11 to 1.57)	
70-79	1.65 (1.34 to 2.02)		1.81 (1.46 to 2.24)	
≥80	2.39 (1.82 to 3.13)		2.72 (2.06 to 3.61)	
Townsend deprivation fifth:				
1 (least deprived)	1 (reference)	NS	1 (baseline)	NS
2	1.02 (0.88 to 1.19)		0.96 (0.82 to 1.12)	
3	1.03 (0.89 to 1.19)		0.97 (0.83 to 1.13)	
4	1.03 (0.89 to 1.19)		0.94 (0.81 to 1.09)	
5 (most deprived)	0.94 (0.81 to 1.10)		0.81 (0.69 to 0.95)	
Missing	1.21 (0.96 to 1.52)		1.1 (0.87 to 1.39)	
Neuropsychiatric diagnosis:				
Severe mental illness	8.87 (7.76 to 10.15)	<0.001	6.69 (5.83 to 7.68)	<0.001
Challenging behaviour	2.51 (2.30 to 2.76)	<0.001	2.08 (1.90 to 2.27)	<0.001
Autism	1.87 (1.66 to 2.12)	<0.001	1.79 (1.56 to 2.04)	<0.001
Depression	2.49 (2.27 to 2.72)	<0.001	1.79 (1.62 to 1.98)	<0.001
Anxiety	2.36 (2.15 to 2.60)	<0.001	1.63 (1.47 to 1.81)	<0.001
Dementia	1.91 (1.54 to 2.40)	0.003	1.42 (1.12 to 1.81)	0.003
Epilepsy	1.04 (0.94 to 1.15)	NS	1.15 (1.04 to 1.28)	0.007

IRR=incidence rate ration; NS=not significant.

*Univariable analysis.

†Multivariable analysis adjusted for age, sex, Townsend deprivation fifth, neuropsychiatric diagnoses, and year of study entry.

Figure 3 shows the overlap between people with a record of severe mental illness, challenging behaviour, and prescription of antipsychotics. Of 9135 peoples treated with antipsychotic drugs by the end of follow-up, 6503 (71%) did not have a record of severe mental illness. Of the 11 915 with a record of challenging behaviour, 5562 (47%) had received antipsychotic drugs, whereas only 1421 (12%) had a record of severe mental illness. Of those with a record of prescription of antipsychotics, 2362 (26%) did not have a record of severe mental illness or challenging behaviour. Further detail of the overlap between challenging behaviour, prescription of antipsychotics, and neuropsychiatric diagnoses is given in web appendix B.

**Figure fig3:**
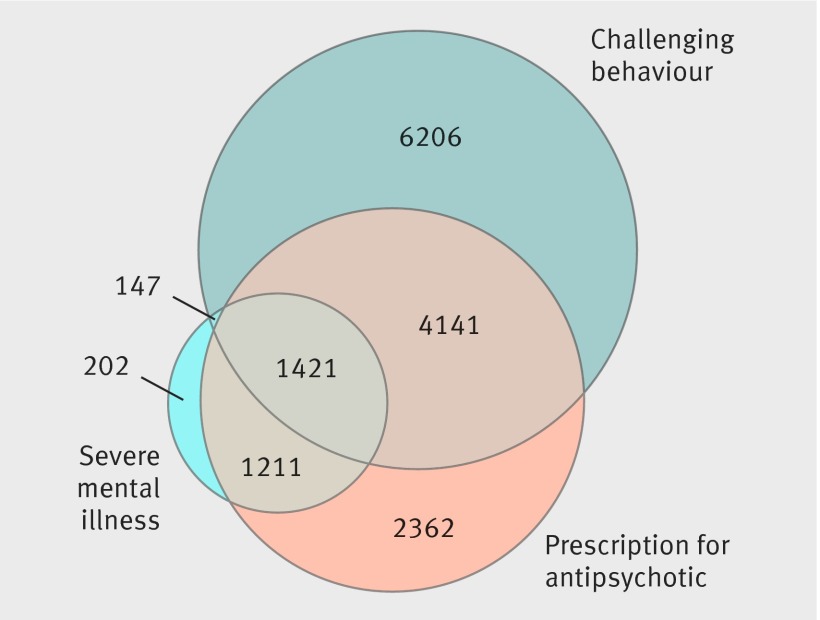
**Fig 3** Relations between recorded severe mental illness, challenging behaviour, and prescription of antipsychotic drugs in adults with intellectual disability

## Discussion

To the best of our knowledge, this study represents the largest and most comprehensive analysis of the associations between mental illness, challenging behaviour, and prescribing of psychotropic drugs in people with intellectual disability. We found that more than a third of people with intellectual disability had a record of mental illness, including almost 1 in 10 who had a record of severe mental illness. Challenging behaviour was also commonly recorded by general practitioners. The proportion of people who had been treated with psychotropic drugs was much greater than the recorded rate of psychiatric morbidity. More than two thirds of people had a record of prescription of any psychotropic drug, and more than a quarter had received an antipsychotic. Most antipsychotics were prescribed to people without a record of severe mental illness, despite financial incentives to record mental illness via the QOF scheme.

Challenging behaviour was independently associated with prescription of antipsychotics after adjustment for neuropsychiatric diagnoses, suggesting that these drugs are being used to manage challenging behaviour in primary care in some cases. Autism, dementia, and older age were also independently associated with antipsychotic use in people with intellectual disability.

We found that the recording of new severe mental illness and the prescription of antipsychotics and mood stabilisers to people with intellectual disability in primary care decreased consistently over the past 15 years, whereas rates of common mental disorders and challenging behaviour, along with other classes of psychotropic drugs, did not change.

### Comparison with other studies

We used routine primary care records to determine the incidence of newly recorded mental illness and challenging behaviour in people with intellectual disability, so our results are not directly comparable with previous epidemiological studies, most of which report the point prevalence of morbidity after direct assessment of participants’ mental state. Such studies consistently report high rates of severe mental illness in people with intellectual disability.^26^ ^27^ The incidence of newly recorded severe mental illness that we found in people with intellectual disability (32 per 10 000 person years) is substantially higher than that in the general population (reported by a different study but derived from the same database^28^), suggesting that severe mental illness is generally well recognised in people with intellectual disability in primary care. In contrast, we did not find increased rates of new recording of anxiety and depression in people with intellectual disability over the study period compared with the general population,^29^ ^30^ despite evidence that they are likely to be at higher risk of developing both conditions.^31^ ^32^ Our results may therefore indicate under-recognition of depression and anxiety in people with intellectual disability in primary care.

Longitudinal trends in recording of mental illness in primary care have not previously been studied in people with intellectual disability, and it is interesting that the rate of recording of new cases of severe mental illness steadily decreased. Several possible explanations for this observation exist. It might mirror a trend highlighted in the non-intellectual disability population whereby clinicians in primary care increasingly use Read codes for symptoms rather than diagnoses to record mental illness.^29^ ^30^ The symptoms would not be recognised in our code lists as severe mental illness. Alternatively, the trend may be due to increasing under-recognition of severe mental illness in people with intellectual disability, although the relatively high crude incidence rates we found suggest that this is not the case. Another explanation would be that the true incidence of severe mental illness is falling, possibly owing to improvements in public health that have reduced precipitating factors.

This is the first study of which we are aware that has quantified challenging behaviour in primary care by using routinely collected health data. We found that challenging behaviour was recorded in more than a third of people, a rate that is higher than the 10-15% previously reported in people with intellectual disability.^3^ However, considerable discrepancy exists in previously reported rates of challenging behaviour in people with intellectual disability owing to differences in definitions and groups studied. The expected associations of challenging behaviour with degree of intellectual disability, autism, mental illness, and dementia were replicated in our study,^33^ ^34^ ^35^ ^36^ ^37^ and incidence rate ratios for each were similar to those reported by a recent population study in Sweden,^38^ which adds legitimacy to the code list we devised. Furthermore, the positive associations between challenging behaviour and degree of intellectual disability, as well as the higher rates in people with autism compared with Down’s syndrome, were as expected. We found an unexpected increase in new cases of challenging behaviour with age,^3^ ^33^ ^38^ ^39^ which persisted even when we controlled for increased recording of dementia among the older population. Dementia can be difficult to diagnose in people with intellectual disability and may be under-recognised^40^; if all true cases of dementia were coded, the association of challenging behaviour with age may be less marked. Another explanation is that the code list for challenging behaviour we devised might include codes that are preferentially applied to the older population.

At 132 per 10 000 person years, the rate of prescription of antipsychotics in people with intellectual disability is almost twice that of the general population (70/10 000 person years for women and 61/10 000 person years for men, from previously published research using THIN data^41^). Approximately 50% of prescriptions for antipsychotics in primary care to people without intellectual disability are given in the absence of a record of severe mental illness,^41^ and our findings show an even higher proportion (71%) in those with intellectual disability. This finding is comparable to that of a Norwegian study, which reported that only 24% of antipsychotics prescribed to a group of people with intellectual disability were indicated by a diagnosis of psychosis,^42^ and other European work showing that most antipsychotics prescribed to people with intellectual disability are given to manage behavioural problems rather than mental illness.^43^ However, the results contrast with a more recent North American survey, which reported that only a minority of antipsychotic drugs were prescribed to people without a formal psychiatric diagnosis.^44^ That prescription of antipsychotic and mood stabilising drugs in people with intellectual disability reduced consistently over the past 15 years is at variance with trends in the use of psychotropic drugs reported in the general population and among adults with intellectual disability in Taiwan.^9^ ^10^ ^45^ The fall in prescription rates that we have shown might be secondary to reduced incidence or recognition of severe mental illness or might reflect a gradual change in practice away from using psychotropic drugs as criticism of perceived overuse has intensified.^46^ ^47^

### Strengths and limitations

The THIN database is a record of real life clinical practice in UK primary care and thus provides an accurate and representative insight into contemporary care. Use of routinely collected data has advantages for research in people with intellectual disability, who may be difficult to reach and enrol in clinical trials or observational studies.^48^ As well as small sample sizes, active recruitment may result in an over-representation of higher socioeconomic groups or people with more engaged carers, which may not be representative of the general intellectual disability population. Furthermore, investigations in the secondary care setting may tend to enrol more severe cases with more challenging behavioural and psychiatric problems and thus lead to overestimates of the use of psychotropic drugs in people with intellectual disability.

Many of the limitations of the study are inherent to the use of routine health data. Using our code list, we estimated a prevalence of intellectual disability of 0.9% in THIN data, which is similar to population estimates.^20^ The slightly lower prevalence in our data may reflect the exclusion of young people, the underdiagnosis of milder cases by general practitioners, or the inclusion of lower income countries in the meta-analysis. We may have missed a small proportion of mild cases of intellectual disability, which if included and assuming that the behavioural problems in this group are generally less challenging, may have reduced the magnitude of the association with prescription of antipsychotics.

The results are of recorded diagnoses, which may not correspond to the true rate of illness in the population. Some diagnoses may have been entered in the free text of the computer system, which we did not interrogate. We restricted our mental illness code lists to the most common mental disorders and excluded categories such as personality disorder and substance misuse; further work is necessary to report on a wider range of illnesses. The challenging behaviour code list has not been externally validated, and we cannot report its sensitivity or specificity. However, it showed the expected associations with conditions known to increase the risk of challenging behaviour and is therefore likely to adequately represent the concept. Certain characteristics, such as degree of intellectual disability, are not always well recorded in THIN, and we have not been able to extend our interpretations to include analysis of these variables. We did not include our own comparison group without intellectual disability and relied on previously published data to put our results in context. Although prescription of drugs is well recorded in THIN, we will have missed the minority of prescriptions issued in secondary care.

We cannot establish with certainty the indication for drug treatment on the basis of THIN data, which may have led to errors in classification where a drug has more than one use (for example, certain mood stabilisers that are also used as anticonvulsants). We infer that in people without a record of severe mental illness and with challenging behaviour, antipsychotics are used to manage behaviour, but this might not be the case. Prescriptions for antipsychotics that do not seem to be supported by a record of severe mental illness are not necessarily inappropriate and may be used within guidelines to treat complex depression or anxiety disorders, for example.

### Clinical implications and future research

Our findings confirm a high rate of prescription of psychotropic drugs to people with intellectual disability in UK primary care and show independent associations of prescribing of antipsychotics with challenging behaviour, autism, dementia, and advancing age. The results suggest that these conditions are managed, in some instances, with antipsychotic drugs, which will often reflect a departure from evidence based clinical guidelines. The finding that the magnitude of the association increased when we excluded sleep disorders from the code list suggests that antipsychotics are not regularly used for sedation at night.

We need to understand why most antipsychotics are prescribed to people without a record of severe mental illness and why so many people with challenging behaviour receive antipsychotics. Limited evidence suggests that certain antipsychotics might be effective in treating behavioural disturbance in adults intellectual disability comorbid with autism,^49^ but no evidence supports antipsychotic use in challenging behaviour outside this context.^7^ Use of antipsychotics for challenging behaviour in people with intellectual disability is recommended only under specialist supervision and for short periods.^24^ Antipsychotics may be used where the availability of other management strategies, such as psychosocial interventions and communication support, is limited. Reducing reliance on drugs will therefore require investment in a skilled multidisciplinary team of professionals who can provide alternative evidence based management strategies for challenging behaviour.

Excessive use of psychotropic drugs has individual and systemic implications. Antipsychotics, in particular, are associated with several adverse side effects that can impair quality of life and lead to deleterious health outcomes.^50^ Reducing the high rate of antipsychotic use in people with intellectual disability might help to reduce the health inequalities that people with intellectual disability experience, which has been identified as a priority area for national action.^51^ Antipsychotic use in people with intellectual disability is complicated by the fact that many people will lack the capacity to consent to taking drugs. We must ensure that patients and their family/carers receive adequate and accessible information on the use of psychotropic drugs and are empowered to question drug treatment and seek alternatives. On a population scale, unnecessary prescribing of drugs burdens health services with avoidable costs, both directly in terms of supplying the drug and indirectly in terms of the additional monitoring that prescription of these drugs mandates.

We concentrated our attention in this study on prescribing of antipsychotics to people with intellectual disability, given that this has been the focus of most controversy. Future research should explore the appropriateness of prescription of other classes of psychotropic drugs in this group. Further investigation of the efficacy and safety of psychotropic drugs is needed, particularly when they are used for challenging behaviour.

### Conclusion

Psychotropic drugs are an important element in the management of specific psychiatric conditions. However, we have shown that adults with intellectual disability are treated with psychotropic drugs at a rate far exceeding that of recorded mental illness and that certain subgroups (such as those with challenging behaviour) are significantly more likely to receive antipsychotic drugs. Although the prescription of antipsychotic drugs has declined over the past 15 years, more work is clearly needed as prescribing often seems to be contrary to the evidence base and clinical guidelines of good practice.

Inappropriate use of drug treatment has implications for the individual and for healthcare systems. Optimising drug use is central to improving care outcomes and will be achieved by a combination of interventions. Adoption of a comprehensive medicines optimisation programme, such as that promoted by NHS England (www.england.nhs.uk/ourwork/pe/mo-dash/), enhanced training of front line professionals, timely recognition and accurate diagnosis of mental illness in people with intellectual disability, and improved accessibility to and development of alternative therapeutic strategies could all contribute to reducing excessive use of psychotropic drugs.

**What is already known on this topic**

People with intellectual disability develop severe mental illness at higher rates than do the general population and may show challenging behaviour
Antipsychotic drugs might be prescribed to people with intellectual disability to manage challenging behaviour, despite lack of evidence and the risk of adverse side effects
The perceived overuse of psychotropic drugs in people with intellectual disability has been widely criticised

**What this study adds**

More than a third of people with intellectual disability have a primary care record of challenging behaviour
Prescription of antipsychotic drugs in UK primary care is disproportionate to the level of recorded severe mental illness and is associated with the presence of challenging behaviour, older age, and diagnoses of autism and dementia
Findings highlight the need for an improved evidence base for use of drugs and optimisation of drug treatment in people with intellectual disability

## Notes

Ethical approval: IMS Health has overall ethical approval to supply data to researchers. The study received approval from the THIN Scientific Review Committee (reference 14-071).

Data sharing: A full list of Read codes used and drugs included is available from the corresponding author.

Transparency declaration: The lead author (the manuscript’s guarantor) affirms that the manuscript is an honest, accurate, and transparent account of the study being reported; that no important aspects of the study have been omitted; and that any discrepancies from the study as planned (and, if relevant, registered) have been explained.

## Notes

Cite this as: *BMJ* 2015;351:h4326
