# Supplementary material for: Mental illness, challenging behaviour, and psychotropic drug prescribing in people with intellectual disability: UK population based cohort study
Source: BMJ. 2015 Sep 1;351:h4326. doi: 10.1136/bmj.h4326 (PMC4556752; doi:10.1136/bmj.h4326)
Supplement: Supplementary file 1 [file sher026514.ww1_default.pdf]

Appendix A – challenging behaviour Read code list

Read code	Description
ZV40.11	[V]Behavioural problems
E2C..11	Behaviour disorder
1B1X.00	Behavioural problem
1B16.00	Agitated
1B16.11	Agitated - symptom
E273100	Head-banging
1469.00	H/O: behaviour problem
1P5..00	Aggressive behaviour
R00zD00	[D]Restlessness and agitation
1BE1.00	Problem situation
E2C0000	Aggressive outburst
1B1I.00	Crying, excessive
1P00.00	Hyperactive behaviour
E2C0100	Anger reaction
E2C1200	Tantrums
E2C..00	Disturbance of conduct NEC
E213.11	Aggressive personality
E274F00	Inversion of sleep rhythm
R00z800	[D]Irritability and anger
1B1O.00	Restless
1B15.11	Irritable - symptom
1B1f.00	Anger
R00zA00	[D]Physical violence
E293000	Adjustment reaction with aggression
ZX17100	Banging own head against object
3AB1.00	Mildly abnormal behaviour
E2Cz.00	Unspecified disturbance of conduct
Ry18.00	[D]Self neglect
E2Cy000	Breath holder
ZX1G.00	Scratches self
ZX1LD00	[X]Self mutilation
2256.00	O/E - agitated
ZV40300	[V]Other behavioural problems
E27z300	Masturbation
E213.00	Explosive personality disorder
E274E00	"Short-sleeper"
1P50.00	Violent acts towards others
1P3..00	Compulsive behaviour
288..11	Uncooperative
14X4.00	On sex offenders register
Eu70y00	[X]Mild mental retardation, other impairments of behaviour
Ry13.00	[D]Overactivity
1B15.00	Irritable
3AB2.00	Severely abnormal behaviour
Eu63300	[X]Trichotillomania

1P51.00	Physically abusive behaviour
1P52.00	Verbally abusive behaviour
1P40.00	Inappropriate shouting
225B.00	O/E - angry
Z4A9.00	Discussion about behaviour
ZX1..00	Self-injurious behaviour
ZX1D.00	Picking own skin
U2E..00	[X]Self mutilation
1P11.00	Uncooperative behaviour
Ry12.00	[D]Strange and inexplicable behaviour
14XA.00	Alleged perpetrator of sexual abuse
ZX11.11	Bites self
13HN000	Theft
13Z8000	Social adjustment problem
E216.12	Dependent personality
E216.13	Labile personality
E275200	Pica
E275711	Compulsive water drinking
E2Czz00	Disturbance of conduct NOS
E27z400	Nail-biting
Eu72100	[X]Severe mental retard sig impairment behaviour requiring attention/treatment
J08z100	Lip biting
ZX1P.00	Swallowing substances
1P02.00	Disinhibited behaviour
1Q0..00	Impulsive character
E2Cy.00	Other conduct disturbances
Eu70100	[X]Mild mental retard sig impairment behaviour requiring attention/treatment
Eu72y00	[X]Severe mental retardation, other impairments of behaviour
ZX11500	Biting own tongue
1P53.00	Argumentative behaviour
E2E2.00	Hyperkinetic conduct disorder
28L..00	O/E - impulsive behaviour
14XC.00	Alleged perpetrator of domestic violence
ZX14200	Pulling out sutures
ZX11.00	Biting self
E27z000	Hair plucking
Eu71100	[X]Moderate mental retard sig impairment behaviour requiring attention/treatment
Eu7zy00	[X]Unspecified mental retardation, other impairments of behaviour
Eu92.11	[X]Emotional behavioural problems
Eu7yy00	[X]Other mental retardation, other impairments of behaviour
ZX17200	Banging own head against own body parts
13HN100	Shoplifting
ZX1..14	Self-abusive behaviour
ZX17.00	Head-banging
ZX1O.00	Swallowing objects
Eu63100	[X]Pathological fire-setting
ZX1..11	Self-destructive behaviour
ZX14.00	Damaging own wounds

ZX17300	Head-butting other person
ZX19.00	Hitting self
ZX19300	Head-hitting
ZX1F.00	Rubbing own skin
14X9.00	Alleged perpetrator of physical abuse
E293100	Adjustment reaction with antisocial behaviour
Eu71y00	[X]Moderate retardation with other behavioural impairment
J08z000	Cheek biting
R00z700	[D]Demoralization and apathy
R2y3.11	[D] Self neglect
ZX1..12	SIB - Self-injurious behaviour
28C..00	O/E - embarrassing behaviour
E293.00	Adjustment reaction with predominant disturbance of conduct
Eu7z100	[X]Unspecified mental retard sig impairment behaviour requiring attention/treatment
R00z900	[D]Hostility
ZX11200	Biting own fingers
ZX11400	Biting own arm
ZX16211	Poking own eyes
ZX19200	Slapping self
E2C1100	Solitary stealing
E2C4.00	Mixed disturbance of conduct and emotion
E2C4z00	Mixed disturbance of conduct and emotion NOS
Eu73100	[X]Profound mental retard sig impairment behaviour requiring attention/treatment
Eu7y100	[X]Other mental retard sig impairment behaviour requiring attention/treatment
Z7CCC00	Found wandering the streets
ZX11100	Biting own hand
ZX11111	Hand biting
ZX11300	Biting own toes
ZX16300	Gouging own rectum
ZX1L.00	Self-mutilation
E2C3300	Pyromania
Eu73y00	[X]Profound mental retardation, other impairments of behaviour
Z7CCB00	Wandering
ZX19100	Punching self
ZX1A.00	Inserting foreign bodies into own orifices
ZX1E.00	Pinching self
ZX1L100	Self-mutilation of hands
ZX1S.00	Throwing self onto floor
14XB.00	Alleged perpetrator of emotional abuse
1B1M.00	Resents authority
1P10.00	Clinging behaviour
28C..11	O/E - irreverent behaviour
E213.12	Quarrelsome personality
E275111	Compulsive eating disorder
E292y00	Adjustment reaction with mixed disturbance of emotion
E292z00	Adjustment reaction with disturbance of other emotion NOS
E293200	Adjustment reaction with destructiveness
E293z00	Adjustment reaction with predominant disturbance conduct NOS

E294.00	Adjustment reaction with disturbance emotion and conduct
E2C3400	Intermittent explosive disorder
E2C3500	Isolated explosive disorder
E2Cyz00	Other conduct disturbances NOS
E2D1.13	Taunting / tormenting
Z43C.00	Questioning the client about his or her behaviour
Z43C.11	Challenging the client about his or her behaviour
Z43G800	Smacking
ZX11600	Biting sides of own cheeks
ZX14100	Reopening scars
ZX14300	Poking fingers into wound
ZX14400	Inserting objects into wound
ZX16.00	Gouging own body parts
ZX16100	Gouging own flesh
ZX16200	Gouging own eyes
ZX16400	Gouging own vagina
ZX1C.00	Nipping self
ZX1L200	Self-mutilation of genitalia
ZX1L300	Self-mutilation of penis
ZX1L400	Self-mutilation of eyes
ZX1L600	Self-mutilation of ears
ZX1LA00	Removing own nails
ZX1LB00	Eating own flesh
ZX1LC00	Stripping own skin
14OE.00	At risk of harming others
1BN..00	Wandering
1BN0.00	Wanders during the day
1BN1.00	Wanders at night
1BN2.00	Wanders during the day and at night
E224.00	Exhibitionism
E224.11	Flasher
E22y100	Voyeurism
Eu65000	[X]Fetishism
Eu65200	[X]Exhibitionism
Eu65300	[X]Voyeurism
Eu65y11	[X]Frotteurism
Eu65z11	[X]Sexual deviation NOS
E2C1111	Shop lifting
Z7CC900	Getting lost
Z7CCA00	Getting lost inside the home
1B1B.00	Cannot sleep – insomnia
1B1B.11	C/O – insomnia
1B1B000	Initial insomnia
1B1B100	Middle insomnia
1B1B200	Late insomnia
1B1Q.00	Poor sleep pattern
1BX0.00	Delayed onset of sleep
E274.00	Non-organic sleep disorders

E274.12	Insomnia due to nonorganic sleep disorder
E274000	Unspecified non-organic sleep disorder
E274111	Insomnia NOS
E274200	Persistent insomnia
E274D11	Restless sleep
E274y00	Other non-organic sleep disorder
E274z00	Non-organic sleep disorder NOS
Eu51.00	[X]Nonorganic sleep disorders
Eu51000	[X]Nonorganic insomnia
Eu51200	[X]Nonorganic disorder of the sleep-wake schedule
Eu51y00	[X]Other nonorganic sleep disorders
Eu51z00	[X]Nonorganic sleep disorder, unspecified
Eu51z11	[X]Emotional sleep disorder NOS
Fy0..00	Sleep disorders
Fy00.00	Disorders of initiating and maintaining sleep
Fy02.00	Disorders of the sleep-wake schedule
Fyu5800	[X]Other sleep disorders
R005.00	[D]Sleep disturbances
R005.12	[D]Sleep rhythm problems
R005000	[D]Sleep disturbance, unspecified
R005800	[D]Sleep dysfunction with sleep stage disturbance
R005800	[D]Sleep dysfunction with sleep stage disturbance
R005900	[D]Sleep dysfunction with arousal disturbance
R005z00	[D]Sleep dysfunction NOS
ZV1B100	[V]Personal history of unhealthy sleep-wake schedule

Appendix B – table showing proportion of cohort with each possible combination of challenging behaviour, antipsychotic prescription, and neuropsychiatric diagnosis

Challenging behaviour	Severe mental illness	Anti-psychotic	Autism	Depression	Anxiety	Number in cohort	% of cohort
						12,186	36.91
■						3,218	9.75
■		■				1,853	5.61
			■			1,554	4.71
				■		1,370	4.15
		■				1,230	3.73
					■	997	3.02
■			■			909	2.75
				■	■	860	2.6
■				■		703	2.13
■		■	■			660	2
	■	■				651	1.97
■				■	■	599	1.81
■	■	■				575	1.74
■		■		■	■	495	1.5
■					■	442	1.34
■		■		■		439	1.33
■		■			■	395	1.2
		■	■			343	1.04
		■		■	■	260	0.79
■	■	■		■		249	0.75
■	■	■		■	■	248	0.75
		■		■		229	0.69
	■	■		■		199	0.6
		■			■	192	0.58
■	■	■			■	153	0.46
			■	■		139	0.42
	■	■		■	■	134	0.41
			■		■	133	0.4
■			■		■	124	0.38
■			■	■		122	0.37
■		■	■		■	122	0.37
	■					116	0.35
■	■	■	■			111	0.34
	■	■			■	101	0.31
■			■	■	■	89	0.27
■		■	■	■	■	89	0.27
■		■	■	■		88	0.27
			■	■	■	87	0.26
	■	■	■			78	0.24

						67	0.2
						42	0.13
						39	0.12
						34	0.1
						34	0.1
						32	0.1
						28	0.08
						23	0.07
						23	0.07
						20	0.06
						20	0.06
						18	0.05
						18	0.05
						16	0.05
						14	0.04
						13	0.04
						10	0.03
						5	0.02
						5	0.02
						4	0.01
						3	0.01
						2	0.01
						2	0.01
						2	0.01
